# The Cercal Organ May Provide Singing Tettigoniids a Backup Sensory System for the Detection of Eavesdropping Bats

**DOI:** 10.1371/journal.pone.0012698

**Published:** 2010-09-13

**Authors:** Manfred Hartbauer, Elisabeth Ofner, Viktoria Grossauer, Björn M. Siemers

**Affiliations:** 1 Department of Zoology, Karl-Franzens Universität, Graz, Austria; 2 Sensory Ecology Group, Max Planck Institute for Ornithology, Seewiesen, Germany; Max-Planck Institute of Neurobiology, Germany

## Abstract

Conspicuous signals, such as the calling songs of tettigoniids, are intended to attract mates but may also unintentionally attract predators. Among them bats that listen to prey-generated sounds constitute a predation pressure for many acoustically communicating insects as well as frogs. As an adaptation to protect against bat predation many insect species evolved auditory sensitivity to bat-emitted echolocation signals. Recently, the European mouse-eared bat species *Myotis myotis* and *M. blythii oxygnathus* were found to eavesdrop on calling songs of the tettigoniid *Tettigonia cantans*. These gleaning bats emit rather faint echolocation signals when approaching prey and singing insects may have difficulty detecting acoustic predator-related signals. The aim of this study was to determine (1) if loud self-generated sound produced by European tettigoniids impairs the detection of pulsed ultrasound and (2) if wind-sensors on the cercal organ function as a sensory backup system for bat detection in tettigoniids. We addressed these questions by combining a behavioral approach to study the response of two European tettigoniid species to pulsed ultrasound, together with an electrophysiological approach to record the activity of wind-sensitive interneurons during real attacks of the European mouse-eared bat species *Myotis myotis*. Results showed that singing *T. cantans* males did not respond to sequences of ultrasound pulses, whereas singing *T. viridissima* did respond with predominantly brief song pauses when ultrasound pulses fell into silent intervals or were coincident with the production of soft hemi-syllables. This result, however, strongly depended on ambient temperature with a lower probability for song interruption observable at 21°C compared to 28°C. Using extracellular recordings, dorsal giant interneurons of tettigoniids were shown to fire regular bursts in response to attacking bats. Between the first response of wind-sensitive interneurons and contact, a mean time lag of 860 ms was found. This time interval corresponds to a bat-to-prey distance of ca. 72 cm. This result demonstrates the efficiency of the cercal system of tettigoniids in detecting attacking bats and suggests this sensory system to be particularly valuable for singing insects that are targeted by eavesdropping bats.

## Introduction

Many insects have evolved ultrasound hearing as an adaptation to predation pressure arising from bats that use echolocation for prey localization. Ultrasound hearing and evasive flight maneuvers in response to pulsed ultrasound are highly developed in species belonging to five insect orders: Orthoptera, Mantodea, Coleoptera, Lepidoptera, and Neuroptera [1]. Ultrasound hearing evolved independently in each of these orders and a high diversity of hearing organs is within and among some of the orders (e.g. [2], [3], [4]). Laboratory and field studies suggest that in addition to listening to echolocation calls, the praying mantis *Parasphendale agrionina* may also make use of the wind-sensitive cercal organ for the detection of wind that is generated by an attacking bat [5], [6]. This suggests the cercal organ of insects suffering from bat predation as an additional sensory system for bat detection. Thus insects may detect attacking bats by ears sensitive to ultrasound as well as by their cercal organ.

Acoustic displays of insects are often conspicuous and can be located at great distances, making them excellent for mate attraction [7]. Such displays, however, also reveal the position of singing insects to eavesdropping predators like bats [8], [9]. Considering the broad-band nature of the calling songs of many katydid species detecting echolocation calls, however, may be difficult for singing insects that generate phonatomes (song elements) at a high rate. This makes self-generated auditory masking of echolocation calls likely.

An example of a tettigoniid katydid species generating phonatomes at a high rate can be found in the European *Tettigonia cantans* where males were recently found to suffer from eavesdropping by the lesser and the greater mouse-eared bat, *Myotis blythii oxygnathus* and *M. myotis* [10]. Tettigoniids are not just very sensitive to ultrasound but in addition also possess elaborated cercal appendices covered with wind-sensilla of different length. In this study we therefore test two hypotheses: 1) European Tettigoniid males have difficulty detecting ultrasound pulses played back while they are generating broadband calling songs, and 2) The response of wind-sensitive interneurons of *T. cantans* form the neuronal basis of a backup sensory system able to forewarn insects of attacking bats.

While the evasive responses of many flying acoustic insects to aerial-hawking bats are duly studied, the responses of non-aerial insects to gleaning bats are still not fully understood (for exceptions see: [11], [12]). Gleaning bats that capture prey from surfaces often produce relatively inconspicuous echolocation calls with intensities much below those of aerially foraging bats (e.g. [13]). Substrate gleaning occurs in approximately one-third of all insectivorous bat species [14]. These bats find their prey by detecting and localizing rustling sounds from walking or fluttering insects in vegetation or on the ground [15]–[17]. During approach to rustling prey, even typically “passive listening” bat species like *Myotis myotis* and *M. blythii oxygnathus* continue echolocation throughout the approach phase [18]. In this phase, these bats reduce the amplitude of their echolocation signals, but increase the rate of signal emission [19], [20].

Eavesdropping on insect calling songs has been described for multiple species of bats in the Southern Hemisphere [21], [22], and North America [12], [23], [24]. Recently it was shown that the European mouse-eared bat *Myotis blythii oxygnathus* eavesdrop on calling songs of *T. cantans* and *T. viridissima*, whereas the sibling species *Myotis myotis* eavesdropped only on the calling song of *T. cantans* [10]. Both tettigoniid calling songs are broad-band in nature including ultrasonic signal components that strongly overlap with the hearing range of bats [25], [26]. This facilitates eavesdropping by bats and may in part explain the rich tettigoniid diet of *M. blythii oxygnathus* during summer [27], [28].

Depending on the predation pressure arising from eavesdropping bats, males face a trade-off between the attraction of potential mating partners and the risk of becoming prey of eavesdroppers [7], [9], [29], [30]. Anti-predator strategies of acoustically communicating insects are therefore common and can be classified as either primary or secondary defense mechanisms. Primary mechanisms are typically found in the absence of a direct threat, e.g. a strong reduction of song duty cycle or singing from protected perches [21], [22], [31]. This defense strategy is common in habitats in which foliage-gleaning bats locate their prey by listening to the calling songs of katydids or other prey-generated sounds (Neotropics: e.g. *Micronycteris hirsuta*, *Lophostoma silvicolum*: [21], [32], [33]; North America: e.g. *Myotis septentrionalis*: [34], [35]; Europe: e.g. *M. bechsteinii* and *Plecotus auritus*: [36]–[38]). In addition to bats, parasitoid flies can also eavesdrop acoustic mating displays in order to locate prey. These flies prefer calling songs of field crickets with a longer chirp duration and a higher chirp amplitude [39], [40], a finding that emphasizes the importance of primary defense mechanisms.

After insects detect a hunting predator, secondary defense mechanisms are often initiated. Such responses include song cessation, freezing or escape jumps, and, when in flight, escape flights or a diving response. Such behavior can be found in many katydids [24], [41]–[43], crickets [11], wax moths [24], praying mantids [44] and Neuroptera [45]. In some orthopterans, cessation of calling has been described as a secondary anti-predator startle response that can be elicited by presenting pulsed ultrasound, which mimics the echolocation calls of attacking bats [12], [22], [24], [46]–[50]. The effectiveness of an acoustic startle response in the form of song cessation was recently demonstrated for *Neoconocephalus ensiger*. In this insect, song cessation interrupts attacks of the gleaning bat *Myotis septentrionalis* [51].

Field crickets are able to discriminate ultrasound signals of bats from conspecific calling songs in the frequency domain [52]. Such a categorical perception of conspecific signals and predator-related signals is impossible for many katydids because calling songs are broadband signals that extend well into the ultrasonic frequency range where they overlap with echolocation signals of bats (>60 kHz: [21], [25], [53]). Nevertheless, a discrimination of echolocation signals from conspecific calling songs seems to be possible in the time domain [43], [54], [55]. In addition to the problem of discriminating conspecific signals from predatory signals, singing orthopterans face another problem that results from a small distance between the site of sound generation and hearing organs. Self-generated sound pressure levels can be very high, a fact that favors auditory masking of echolocation calls emitted by hunting bats. Indeed, Faure and Hoy [49] revealed that acoustic startle responses in the form of song cessation and song pausing in *Neoconocephalus ensiger* is restricted to windows of silence when the pulse of ultrasound arrives between stridulatory syllables. Also in field crickets a stridulatory phase dependent shielding of the hearing system from self-generated sound was found [56]. There a corollary discharge mechanism suppresses auditory receptor activity and inhibits acoustic neurons from firing during syllable production.

Some tettigoniids generate phonatomes at a very high rate and a similar protective mechanism as was found in field crickets may strongly impair the acoustic detection of echolocation calls. The sympatrically occurring European tettigoniid species *T. cantans* and *T. viridissima* can be clearly distinguished by the temporal structure of their calling songs. *T. cantans* produces verses of increasing amplitude exhibiting a monosyllabic rhythm with no pauses of more than 3 ms between adjacent syllables. In contrast, the calling song of *T. viridissima* is even louder and consists of two hemi-syllables, which are grouped into a disyllabic rhythm by ∼18 ms pauses. For recognition of conspecific calling songs, female *T. viridissima* assess the syllable-pause structure rather than the double-syllable rate [57]. Due to this differing temporal song structure of both tettigoniid species, a response to ultrasound pulses may be more likely elicited in *T. viridissima* males.

Singing insects whose ability to detect sound is impaired by self-generated acoustic signals may rely on a sensory backup system for the detection of approaching bats. The wind-sensitive cercal system might function as such a backup because in praying mantis wind generated by approaching bats elicits a neuronal response [5]. The role of this cercal organ in bat evasion is further supported from observations of free-flight encounters between praying mantis and bats [6]. Wind-sensilla also play an important role in the escape behavior of cockroaches (e.g. [58]) or firebrats [59]. Similarly, crickets are capable of detecting and acting evasively toward the airborne disturbances created by wing beats of a flying parasitoid wasp (up to 3 cm away [60], [61]). The role of the cercal system for predator detection is as yet unaddressed in tettigoniids, however. We therefore studied the response of wind-sensitive interneurons of *T. cantans* during the approach of the greater mouse-eared bat *Myotis myotis*. This method is similar to the study of Triblehorn and Yager [5] who showed that the early response of wind-sensitive interneurons gives the praying mantis *Parasphendale agrionina* 36 ms for an escape response.

Our study combines a behavioral approach to song-pausing induced by ultrasonic signals with an electrophysiological approach in order to investigate the role of the cercal system in detecting approaching bats. Our aim was to test whether (1) tettigoniids impair their acoustic bat detection ability by their own singing with a higher probability of song pausing in *T. viridissima* and (2) whether the cercal system may function as a backup to detect approaching bats by means of mechano-sensors while an acoustic detection is impaired.

## Results

### Sound pressure level of singing insects

The sound pressure level measured in peak hold function in one cm distance to the front leg of singing males was 120±1.5 dB SPL (N = 6) for *T. viridissima* and 113±3.2 dB SPL (N = 4) for *T. cantans* respectively. Switching from peak level measurement to fast integration mode reduced peak SPL by 13 dB. It needs to be considered that for frequencies lower than 16 kHz the microphone was in the near field as a result of only two cm separating the microphone and the stridulatory apparatus of males.

### Ultrasound stimulation of singing *T. cantans* males

**Figure 1:**
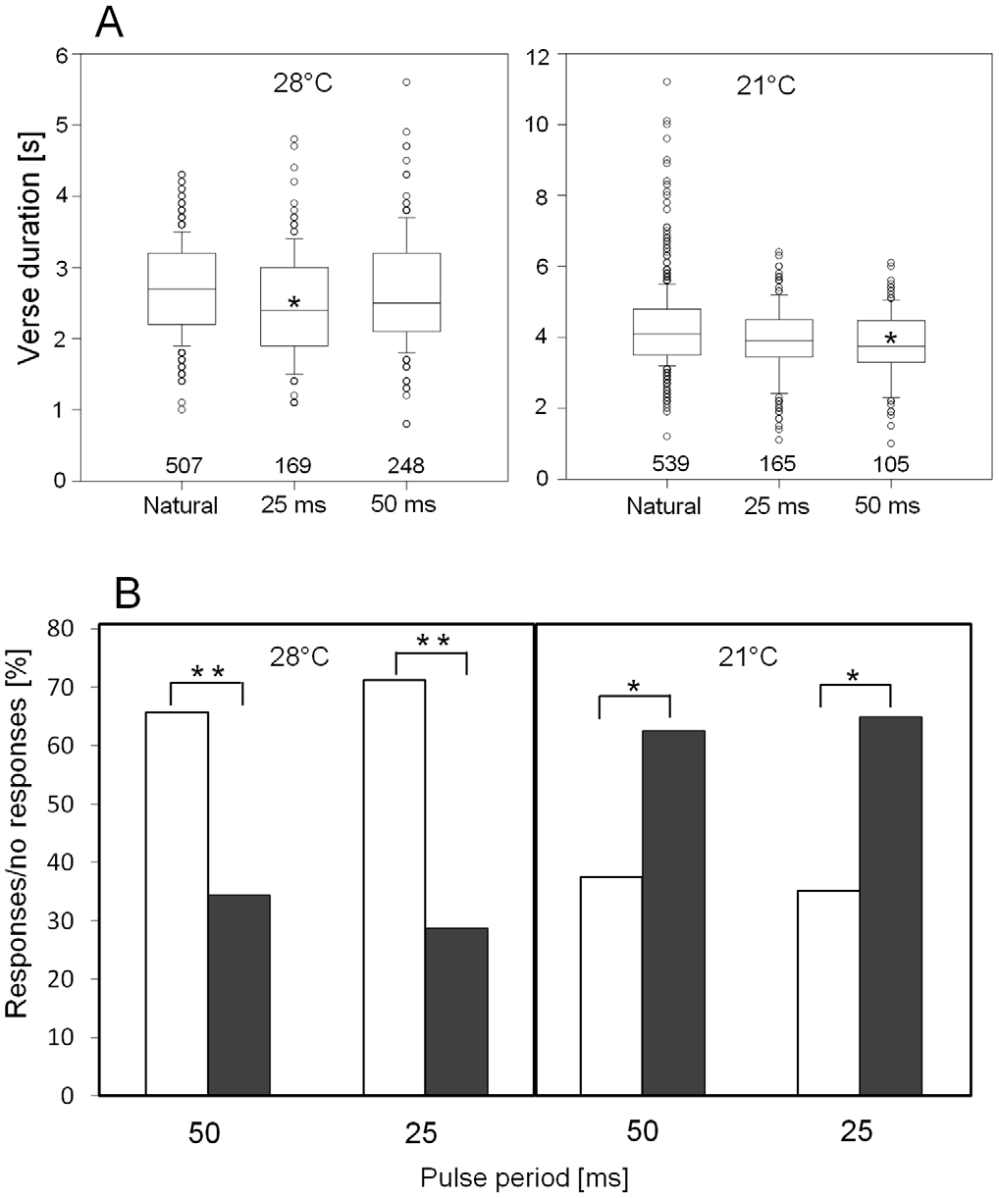
Response to playback of repetitive ultrasound pulses to singing tettigoniids. (A) Average verse duration of *T. cantans* males with and without repetitive ultrasound stimulation (25 ms and 50 ms pulse repetition period). Numbers in A represent the number of verses in each group. A significant difference (p<0.05) of stimulated verse durations from natural verse durations is indicated by *. B) Response of *T. viridissima* males to ultrasound stimulation in the form of song pausing (white bars) and the lack of such a response (black bars). 28°C: N = 131 (50 ms), N = 115 (25 ms); 21°C: N = 40 (50 ms), N = 38 (25 ms). All data was obtained from 8 *T. cantans* and 7 *T. viridissima* males. p<0.05 is indicated by *, p<0.001 is indicated by **; Upper and lower box limits in A represent 25 and 75 percentile and whiskers 10 and 90 percentile. Outliers are indicated by circles.

**Figure 2:**
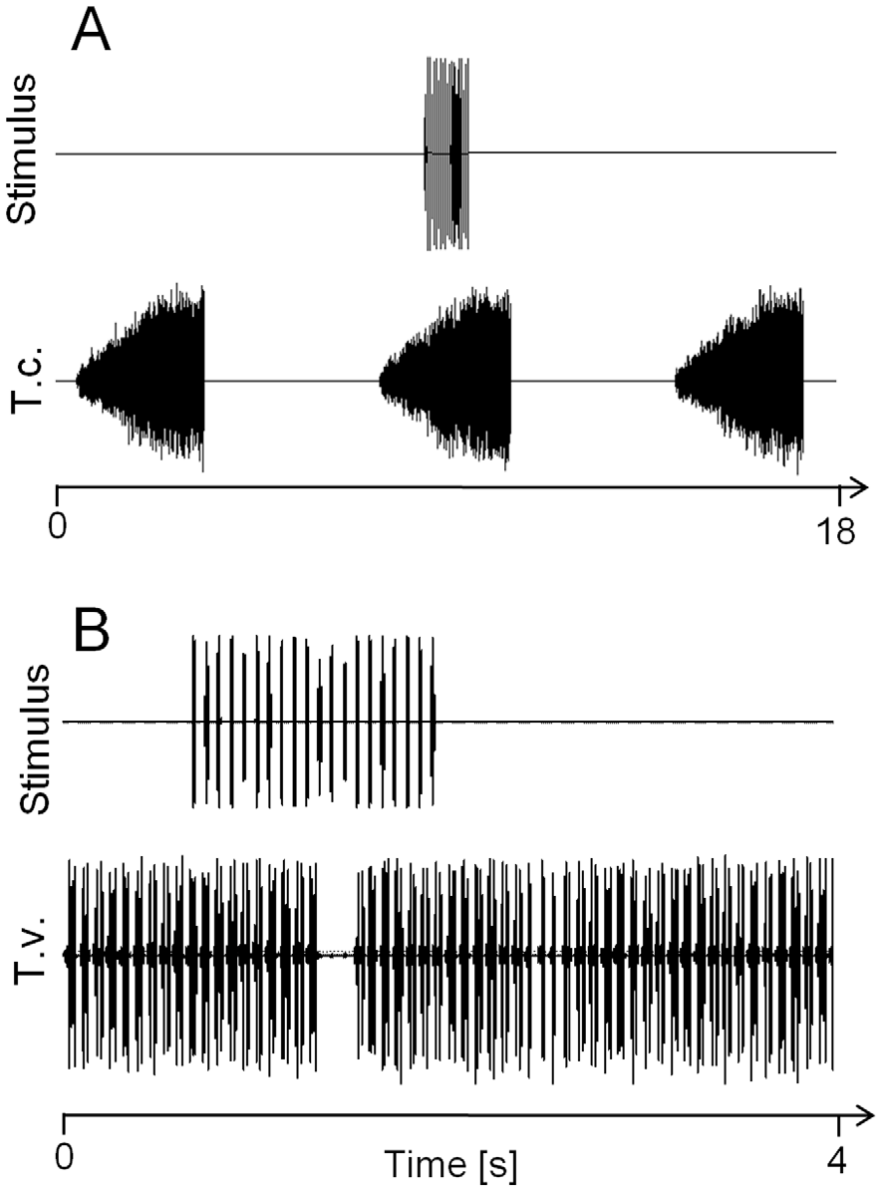
Examples of ultrasound stimulation of *T. cantans* and *T. viridissima* males. (A) *T. cantans* males were stimulated with repetitive 30 kHz pulses during verse production. The same stimulus frequently elicited brief song pausing in *T. viridissima* (B). Stimulus level was 89 dBpe SPL.

The syllable rate as well as verse duration of *T. cantans* songs were temperature dependent. Verses of *T. cantans* males were significantly longer at 21°C compared to 28°C (compare “Natural” in figure 1A). The pause duration between phonatomes was very short and only slightly affected by ambient temperature (2.8 ms at 28°C vs. 3.6 ms at 21°C). After presenting a sequence of ultrasound pulses during singing, males rarely stopped singing while the stimulus was running (see example in figure 2A). However, due to the high variability of verse duration it was impossible to distinguish between natural verse pausing and stimulus-induced pauses. Nevertheless, if a sequence of ultrasound pulses leads to verse pausing, the average length of undisturbed verses should be longer compared to verses coinciding with ultrasound stimulation (stimulated verses). However, an analysis of verse durations calculated across males revealed only a very small reduction of 200–350 ms in stimulated verse duration compared to natural (undisturbed) verses (Fig. 1A). This difference between stimulus groups and controls was statistically significant for a pulse period of 25 ms tested at 28°C and 50 ms tested at 21°C (see asterisks in figure 1A). A comparison of natural and stimulated verse durations including male individuals as a separate factor showed that only one out of eight males significantly shortened its verses following stimulation with ultrasound pulses (p<0.05, Two-way ANOVA on ranks followed by a Dunn's post hoc test). This result was the same at both temperatures.

### Song pausing of *T. viridissima* males

Starting in the afternoon, *T. viridissima* males produced long lasting song bouts with an average length of 133±11 min at 28°C and 109±11 min at 21°C (N = 7). Song bouts were interrupted by infrequent natural pauses of 200±1200 ms average duration. Natural pauses lead to a stuttering that occurred on average every 152.3 s, which was rare enough in order to prevent confusion with song pausing elicited by a sequence of ultrasound pulses (see example in Fig. 2B). The frequency of stimulus-induced song pausing (response) was strongly dependent on ambient temperature. At both tested temperatures the difference in the proportion of responses (song pausing) and no responses was found to be significantly different. However, males singing at 28°C interrupted their songs in response to a sequence of ultrasound pulses almost twice as often as males singing at 21°C (white bars in figure 1B).

The difference in the frequency of song pausing at a lower temperature could be a consequence of slower stridulatory movements changing temporal song parameters (summarized in Table 1). Indeed, the duration of double-syllables was significantly prolonged at lower temperatures. In contrast, the average pause duration separating pairs of syllables was only slightly longer at 21°C compared to 28°C (22 ms vs. 18 ms). This resulted in a reduced syllable rate of only 13 Hz at an ambient temperature of 21°C.

**Table 1:** Temperature influence on song parameters of *T. viridissima* males.

Temp.	Double syllable period	Double syllable duration	Pause between syllable pairs	Syllable rate	Duty cycle	Song bouts evaluated
°C	ms	ms	ms	Hz	%	N
21	76.7±1.44	54.7±3.1	22.0±1.6	13±1.0	71.3	233
28	58.2±0.55	40.0±0.79	18.1±0.24	17.2±0.1	68.8	590

*T. viridissima* males tested at 28°C paused their songs significantly longer in response to a sequence of ultrasound pulses compared to the duration of natural pauses (Table 2). Pulse repetition rate had no significant influence on the duration of song pausing. After presentation of a sequence of ultrasound pulses with a pulse period of 50 ms *T. viridissima* males paused their songs with a latency of 268±235 ms at 28°C. Here, latency is defined as the time lag between stimulus onset and the onset of a song pause. Compared to a pulse period of 50 ms song pausing occurred with a significantly shorter latency of only 209±167 ms in response to the “25 ms stimulus” (Table 2).

**Table 2:** Detailed analysis of song pausing in *T. viridissima* (28°C).

Ultrasound stimulation	Duration of song pausing	Mann Whitney test	Pulse repetition rate	Duration of song pausing	ANOVA	Latency	Mann Whitney test
	s	p	ms	s	p	ms	p
Yes	0.29±0.29 (N = 297)	0.001	50	0.32±0.4 (N = 166)	0.072	268±235 (N = 166)	0.001
No	0.21±0.44 (N = 546)	0.001	25	0.35±0.22 (N = 54)	0.072	209±167 (N = 54)	0.001

### Stridulatory-phase dependency of song pausing

**Figure 3:**
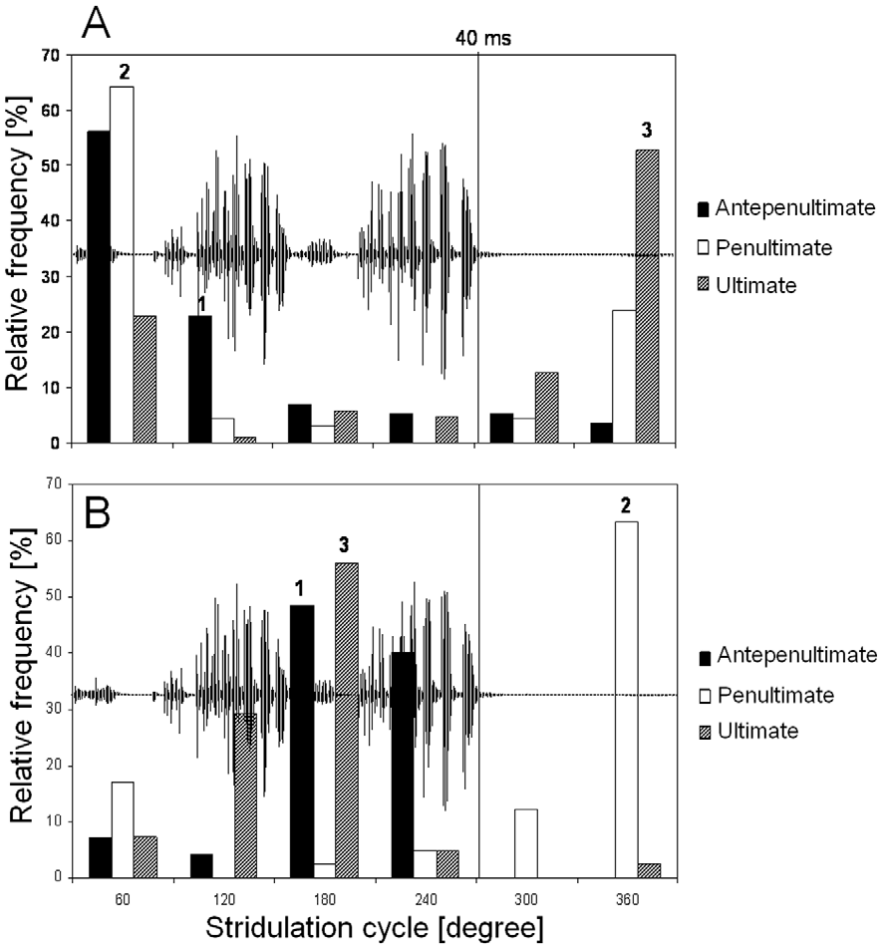
Phase-dependent song pausing of *T. viridissima* males elicited by repetitive ultrasound pulses. Histograms showing the phase of ultrasound pulses preceding song pausing in the stridulation cycle of *T. viridissima*. (A) 50 ms pulse repetition period: Two of three consecutive ultrasound pulses (labeled 2, 3) preceding song pausing were coincident with phases of relative silence. (B) The majority of ultrasound pulses presented with a pulse repetition period of 25 ms coincided with pauses between double-syllables (labeled 2) as well as with soft hemi-syllables separating syllable pairs (labeled 1,3) (B). Vertical lines in A and B indicate the average double syllable duration (40 ms). Data shown in histograms were obtained from 131 stimulus-associated song pauses (7 males tested at an ambient temperature of 28°C).

The startle response of *T. viridissima* males to a sequence of ultrasound pulses was studied by plotting the occurrence of the last three ultrasound pulses eliciting song pausing in the phase of the stridulatory cycle. A histogram display of stimulus phases revealed that males preferentially paused their songs when at least two ultrasound pulses were coincident with the production of soft hemi-syllables or fell into pauses separating double-syllables (Fig. 3). For a pulse period of 50 ms this phase-dependent response was further investigated by categorizing responses as hits and misses depending on the occurrence of ultrasound pulses in the stridulation cycle. Hits represent song pauses that were preceded by two successive pulses coinciding with the production of soft hemi-syllables and between-syllable pauses (< = 60° and >240°C). Missed signals represent pulse pairs that occurred in the same range of stridulatory phases, but failed to elicit song pausing. Classifying pulse pairs in this manner resulted in about twice the number of hits compared to missed pulse pairs (Table 3). For control purposes the same data set (131 stimulus related song pauses) was scanned for single pulses that occurred in that range of stridulatory phases committed to the production of loud hemi-syllables (>60 and < = 240°). In this control only 20 hits were found in which a single pulse coincided with the production of loud hemi-syllables before a song pause occurred. In contrast, there were 212 missed signals.

**Table 3:** Stridulatory-phase dependence of song pausing in *T. viridissima*.

Category	Stridulation phase	Hits	Misses	z-test
Phase	degree	N	N	p
Relative silence	< = 60° and >240°	49	21	0.004
Syllable production	>60° and < = 240°	20	212	0.001

A total of 131 stimulus-induced song pauses have been evaluated (7 *T. viridissima* males).

### Wind-evoked nervous response in *T. cantans*

**Figure 4:**
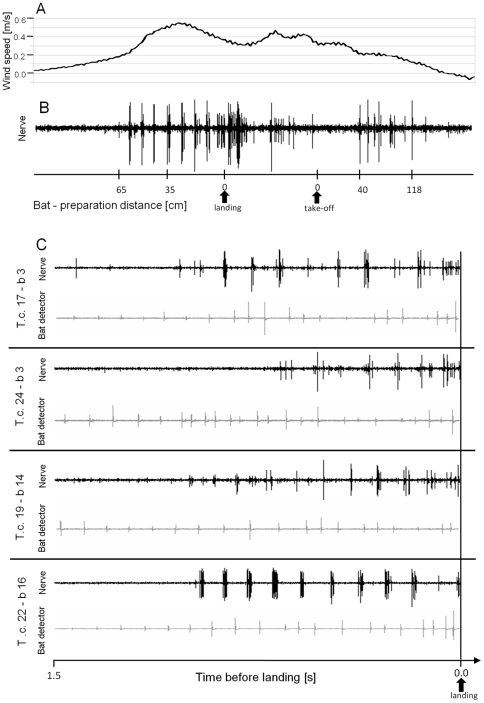
Response of a technical bat detector, an anemometer and wind-sensitive interneurons of *T. cantans* to bat attacks. (A) The wind generated by an approaching bat measured by hot-wire anemometry. (B) The afferent activity of wind-sensitive interneurons in the ventral nerve cord of *T. cantans* was simultaneously recorded by a hook electrode. Three different neuronal units were clearly discernable by means of spike amplitude. (C) The neuronal activity of wind-sensitive interneurons (upper traces) of four different individual insect preparations recorded during bat attacks. Grey traces in C show the simultaneous activity of a technical ultrasound-based bat detector.

The wind velocity generated by bats approaching the insect preparation (*T. cantans*) was measured by means of hot-wire anemometry and resulted in an average maximum wind velocity of 0.58±0.14 m/s (mean of 70 bat approaches, for example see Fig. 4A). Bat approaches were accompanied by a periodic emission of echolocation signals (grey traces in figure 4C) and wing beats elicited regularly occurring bursts fired by wind-sensitive interneurons (Fig. 4B and black traces in 4C). During the approach phase bursts often alternated with the activity of the technical bat detector (compare grey traces with black ones in fig. 4C). According to spike amplitudes three different wind-encoding nervous units were clearly distinguishable (for example see fig. 4B). A bat's landing on top of the preparation caused a characteristic burst of longer duration.

**Figure 5:**
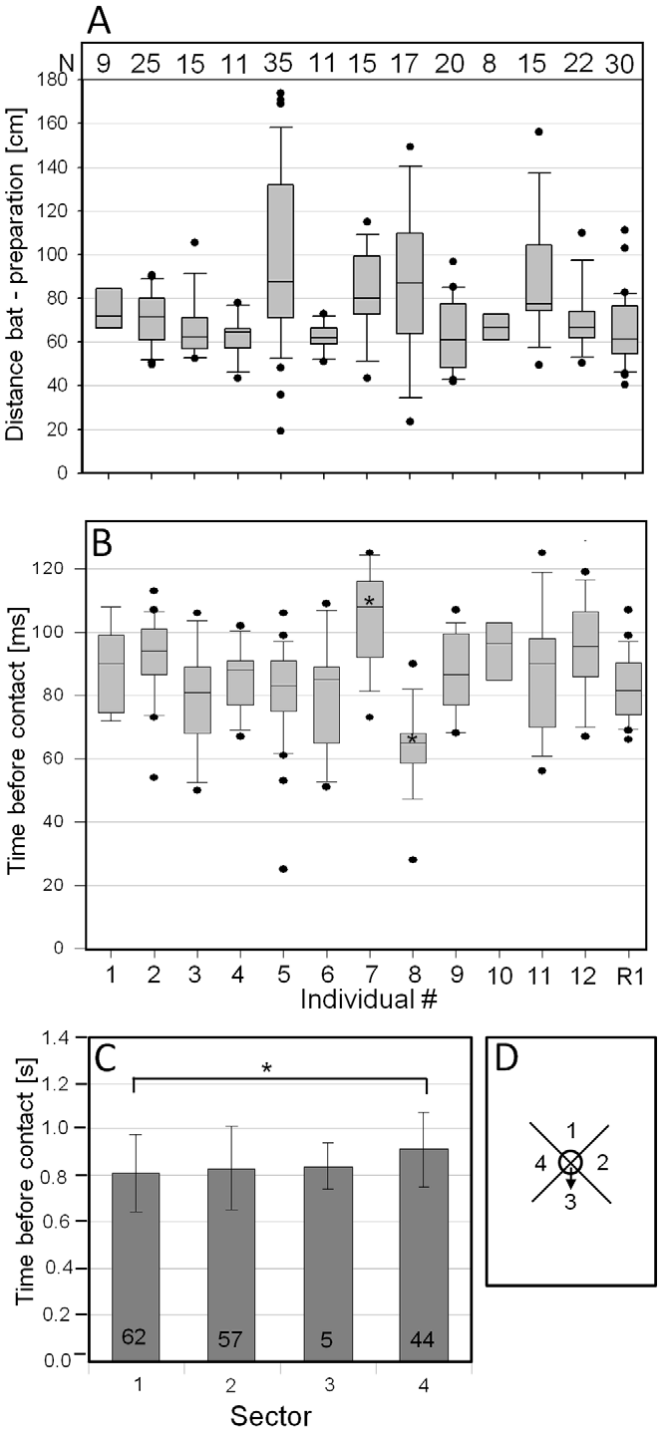
The minimum detection distance and minimum detection time derived from a first neuronal response of wind-sensitive interneurons to approaching bats. (A) The minimum distance between bat and preparation eliciting a first response of wind-sensitive interneurons in 12 individuals of *T. cantans* and one individual of *Ruspolia nitidula* (R1). Numbers in A represent the number of bat approaches. (B) The average minimum time lag between a first nervous response of wind-sensitive interneurons to approaching bats and bat landing (contact with the insect preparation). Asterisks in B indicate p<0.05 which is the result of a two-way ANOVA on ranks followed by a Dunn's post hoc test. (C) A sector-wise comparison of such minimum detection times. Numbers in bars represent the number of bat approaches observed in each sector. * in C indicates p<0.05 as the result of a Kruskal Wallis ANOVA on ranks followed by a Dunn's post hoc test (N = 12 insects). (D) A schematic view of the flight arena and the definition of sectors used in C. The arrow in D indicates the direction of the remaining cercus. For an explanation of box plots see figure 3.

The first neuronal response of wind-sensitive interneurons was observed at a bat-to-preparation distance of 50–160 cm (mean: 72.4 cm) (Fig. 5A). In one trial the first nervous activity was already observed at a distance of 170 cm (individual 5 in figure 5A). From the first nervous activity of wind-sensitive interneurons to contact a mean time lag of 860 ms was measured across 12 *T. cantans* individuals (Fig. 5B). With the exception of individual #7 and #8 the average time lag was not different among all insect preparations. The one individual of Ruspolia nitidula was only different from *T. cantans* individual #7, but not from any other. Interestingly, a spontaneous activity of wind-sensitive units was almost absent (0.93±1.22 spikes per second, average of 12 individual insects).

The flight paths of approaching bats may have an influence on the time lag between the first nervous response and contact. In order to evaluate this possibility, the direction of approaching bats was divided into four even sectors and the time to contact was evaluated for each sector separately. This analysis revealed that for an unknown reason both bats approached insect preparations significantly less often from sector 3, the sector in which the remaining cercus pointed (arrow in Fig. 5D). A sector-wise comparison of lag times showed that bats approaching from sector 4 stimulated wind-sensitive neurons slightly earlier (0.9 s) compared to sector 1 (0.8 s) (Fig. 5C). Lag times of all other sectors were not statistically different from each other.

**Figure 6:**
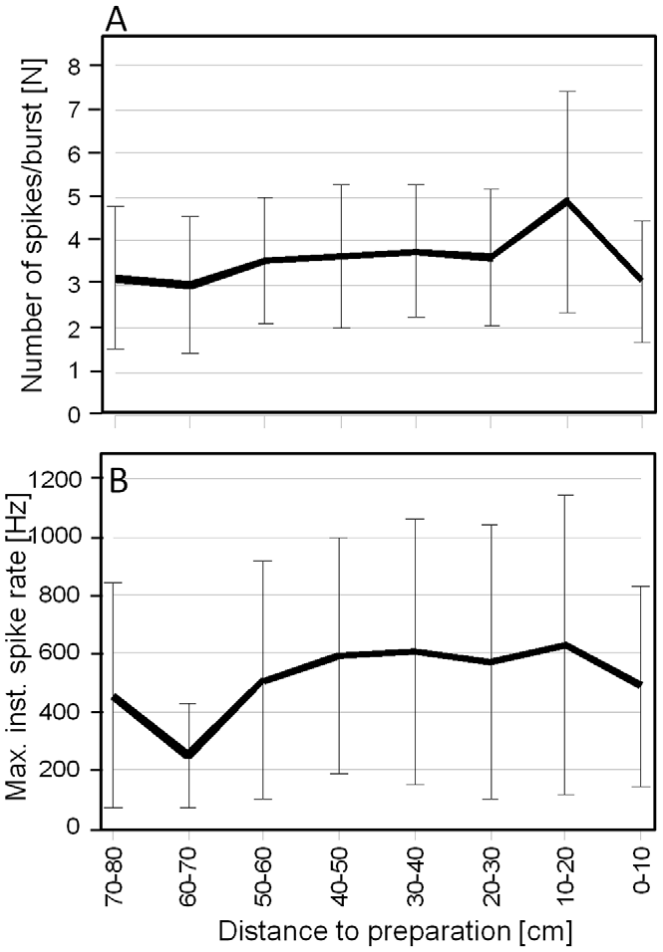
Neuronal response of wind-sensitive interneurons to bat attacks. (A) The average number of spikes per burst fired by all three wind-sensitive interneurons plotted against the distance between bat and insect preparation. (B) The average maximum instantaneous spike rate of a population of three different wind sensitive units. Data were obtained from 52 platform landings (6 *T. cantans* individuals).

Attacking bats elicited distinct bursts in wind-sensitive interneurons with an average spike count of 3–5 (Fig. 6A). Per definition, such bursts were separated by a time interval of at least 20 ms. Considering all three wind-sensitive interneurons as a single unit, a maximum instantaneous firing rate of 250–600 Hz was found (Fig. 6B). Interestingly, mean spike number and mean instantaneous firing rate showed little variation with bat-to-preparation distance.

**Figure 7:**
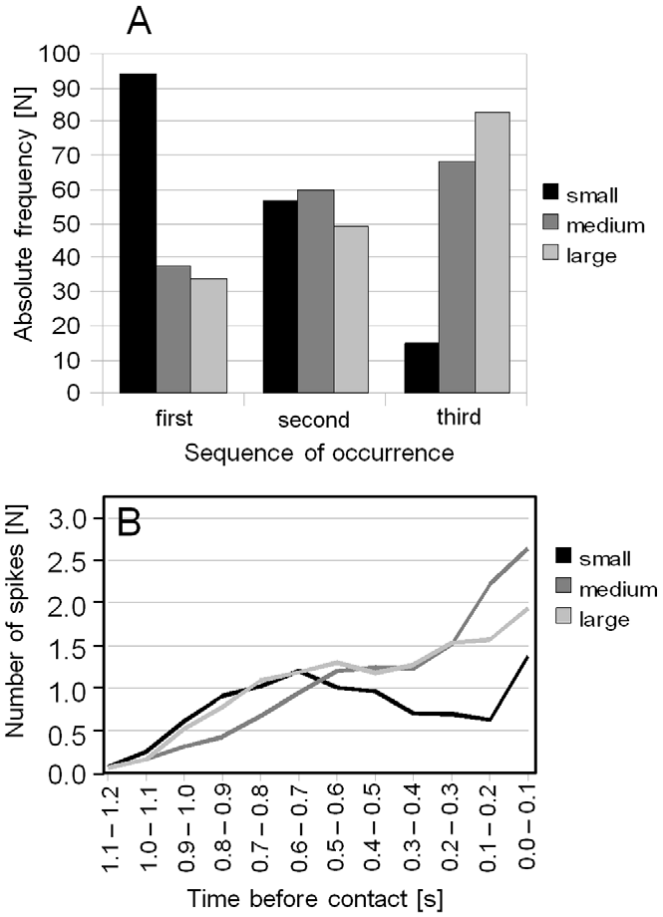
Analysis of the response of three different wind-sensitive interneurons to bats approaches. Three different wind-sensitive interneurons were classified according to their extracellular spike amplitudes into small, medium, large units. (A) The first response of different interneuron classes shown as frequency histogram. Data summarized in A was obtained from 166 platform landings (15 *T. cantans* individuals). (B) The average number of spikes of different wind-sensitive interneurons counted in time windows of 100 ms (125 platform landings obtained from 15 *T. cantans* individuals). A significant negative correlation of the average spike count with bat-to-preparation distance was found for large and medium units (p<0.001, cc = −0.993 (medium), cc = −0.965 (large), N = 12, Spearman Rank Order correlation).

A separate evaluation of all three wind-sensitive units was possible by discriminating extracellular spikes according to their amplitude. This separate analysis revealed a distinct firing sequence: first the small, then medium and finally the large amplitude units (Fig. 7A). However, individual preparations sometimes showed strong deviations from the average firing sequence. Calculation of the average spike count of three different neuronal units in time windows of 100 ms resulted in a gradual increase of spike count of medium and large units during bat approaches (Fig. 7B). A significant negative correlation of the average spike count with bat-to-preparation distance was found for large and medium units, but not for small units. Although small units were the first to respond to an approaching bat (Fig. 7A), on average medium and large units better encode time to contact by spike count (Fig. 7B).

## Discussion

### Detection of pulsed ultrasound by singing tettigoniids

The sensory arms-race between bats and their prey is a classic textbook example in sensory ecology. Acoustically communicating insects face a trade-off between survival and reproduction by producing conspicuous calling songs attracting both intended (potential mates) and unintended receivers (e.g. bats) [7], [10], [29]. A limited detectability of predator-related acoustic cues may place singing males at high risk of becoming prey to eavesdropping bats. The European mouse-eared bat *Myotis myotis* eavesdrop on calling songs of *T. cantans* and the sibling species *M. blythii oxygnathus* eavesdrop on the calling song of both *T. cantans* and *T. viridissima* [10]. Both bat species increase the rate of biosonar signal emission from ∼10 Hz, during the search phase, to ∼25 Hz before prey capture [18]. In this approach phase both bat species significantly decrease the amplitude of their echolocation signals. Compared to the self-generated sound level of both investigated tettigoniids (113–120 dB SPL) the echolocation signals of European substrate-gleaning bats measured at a distance of 1 m appear to be soft (*Myotis myotis* 77.3±4.7 dB peSPL (N = 10), *M. blythii oxygnathus* 80.2±4.0 (N = 12); [62]). Such soft echolocation pulses emitted in the approach phase will be hardly detectable by singing insects, which generate high SPLs in order to advertise themselves to mating partners. Therefore, the situation for singing insects is totally different from resting insects that are able to detect echolocation calls of bats from a great distance (13 m for *Phaneroptera falcata*, [54]).

Playback experiments performed in the current study showed that sequences of ultrasound pulses presented at a SPL typical for gleaning bats resulted in only marginally shorter verse durations generated by *T. cantans* males. Considering the great variability of verse duration, this small reduction would be inadequate as a secondary defense mechanism against gleaning bat attacks (Fig. 2A). Due to the overlap of the spectral content of the calling song of *T. cantans* with the frequency of the ultrasound stimulus and a high phonatome rate, auditory masking of ultrasound pulses is likely. It cannot be excluded that *T. cantans* confuse pulsed ultrasound signals with the calling song of conspecifics. In contrast, a stridulatory-phase dependent response in the form of brief song pauses of *T. viridissima* to pulsed ultrasound was shown for the first time in this study. In this species the detection of ultrasound pulses is restricted to certain phases in the stridulatory cycle (Fig. 3). This phase-dependent response demonstrates the difficulty of *T. viridissima* males detecting pulsed ultrasound during the production of loud hemi-syllables and corroborates results obtained from ultrasound playbacks with *Neoconocephalus ensiger* [49]. Results obtained from playback experiments with both tettigoniids species confirmed hypothesis one of this study.

At an ambient temperature of 21°C, song pausing in *T. viridissima* occurred less frequently compared to 28°C (Fig. 2B). This result is likely due to the temperature affecting the wing stroke rate of the poikilothermic tettigoniids. In both tettigoniids the duration of phonatomes was significantly prolonged at a temperature of 21°C compared to 28°C. In contrast, ambient temperature had little influence on the duration of the silent periods separating consecutive phonatomes. This reduces syllable rate but also the frequency of gaps in which ultrasound can be successfully detected by *T. viridissima* males. Nocturnal temperatures often fall below 21°C and the detection of pulsed ultrasound may be even more difficult for singing *T. viridissima* males compared to what were found in this study. Schul et al. [57] revealed the pause duration separating pairs of syllables in the songs of *T. viridissima* as a critical song parameter for species recognition. A temperature insensitivity of pause duration may thus facilitate species recognition, which, on the other hand, seems to hinder a ready detection of ultrasound pulses with consequences for predator avoidance.

Although flying *Teleogryllus oceanicus* crickets perform a turning behavior away from ultrasound pulses, echolocation signals of gleaning bats did not induce song cessation in males, nor a pause in walking *T. oceanicus* females [63]. This context dependent response could be the result of an adaptation to differing predation pressure for insects in the air as opposed to on the ground. This may also be reflected in the results presented in this study. While *T. cantans* is a non-flying tettigoniid species, *T. viridissima* is a skilled flyer able to perform bat evasive maneuvers during flight [41]. Flying *T. viridissima* expose themselves to a higher risk of predation by aerial hawking bats and a startle response to ultrasound is, therefore, more likely to be found in this species. The surprisingly short duration of song pausing in *T. viridissima* males, however, corroborates results obtained from ultrasound playback experiments performed with male moths that resume signaling after a silent interval of only 100 ms or less [11]. During song pauses tettigoniid males are able to listen to approaching bats. Nevertheless, *T. viridissima* males frequently resumed calling while the ultrasound stimulus was still on (see example in figure 1). This suggests that males may rely on a sensory backup system able to detect approaching bats independently from airborne sound.

### Response of wind-sensitive neurons

The afferent nervous response to the wind generated by an approaching bat resulted in distinct bursts fired by at least three different neuronal units most likely belong to giant interneurons (GINs), which are known to be responsive to wind. Due to their large diameter these neurons generate high amplitude extracellular signals. A bursting response is the consequence of wing beats of approaching bats producing distinct wind puffs. Due to the low frame rate of the video observation system an analysis of a phase-locked response of wind-sensitive neurons to cycles of wing beats was not possible.

The mean instantaneous firing rate of a population of wind-sensitive neurons was in the range of 250 and 600 Hz (Fig. 6B). Assuming that all three wind-sensitive units are targeting the same post-synaptic cell, an immediate response of this cell is likely. Recent studies revealed bursts characterized by short inter-spike intervals of less than 6 ms (166 Hz) to code salient stimulus features and reliably predict behavioral responses [55], [64]–[66]. A high firing rate in the population code of wind-sensitive neurons and the lack of notable spontaneous activity emphasizes a reliable detection of an approaching bat by wind-sensitive sensilla. This enables katydids to perform a last-chance evasive maneuver in the form of an escape jump or escape flight. Both types of response are very likely because the wind on cerci activates flight in tethered locusts and neuronal connections of GINs to the jump motor pathway were found in locusts [67] and in cockroaches [68], [69]. In contrast to ventral GINs, dorsal GINs of cockroaches are more reliable in eliciting motor responses [70]. In *Gryllus bimaculatus* wind puffs directed to the abdominal cerci elicited escape running [71]. Furthermore, in the study of Boyan and Ball [67] wind strength of 1.4 m/s was sufficient to produce a saturated response in the cercal neurons inputting onto the flight motor pathway of locusts. A similar connection of GINs with motor pathways may also exist in katydids where it may enable last-chance escape maneuvers.

Wind-sensitive interneurons of praying mantis first respond to an approaching bat 74 ms before contact [5]. Surprisingly, GINs of *T. cantans* already responded to an approaching bat 850 ms before contact. After subtracting response latency, these katydids have sufficient time left (∼810 ms) for the performance of a life-saving escape response. This result confirms hypothesis two of the current study. However, the minimum detection time for approaching bats mediated through wind sensors will be much shorter compared to the minimum acoustic detection time estimated for resting insects listening to echolocation signals. This needs to be assumed considering a rather low hearing threshold of about 35 dB SPL for ultrasound in katydids [72], [73] and was confirmed for *Phaneroptera falcata* [54]. In a moving predator model of an attacking bat, cockroaches would only have 16–24 ms left for an escape response [74]. This obvious discrepancy in the detection time between *T. cantans* and cockroaches may be explained by differences in the sensory system of these insects, but, more likely, by the fact that the bat model used in Ganihar et al. [74] lacked flapping wings. Furthermore, two wind-sensitive units of tettigoniids showed a graded response to approaching bats (Fig. 7B). This may allow insects at least a rough estimation of the distance to an approaching bat.

**Figure 8:**
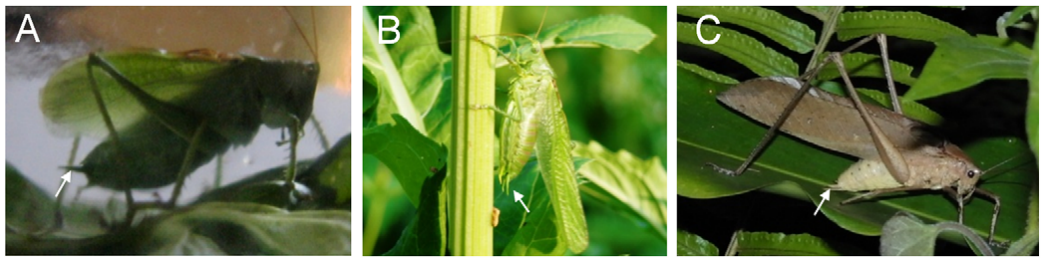
Singing posture of tettigoniids. Singing posture of *T. cantans* (A), *T. viridissima* (B) and *Mecopoda elongata* (trilling species) (C). During singing males lower their abdomen and lift their front wings. Note that this singing posture increases the distance between cerci (arrow) and wings.

Stridulatory wing movements of singing orthopterans may generate wind that could self-stimulate wind-sensilla. Similarly, a flying locust encounters wind speeds of about 5.5 m/s on cerci as a result of its own flight activity [75]. Therefore, a presynaptic inhibition of wind-sensitive filiform afferents by movement-sensitive receptors was suggested, which may allow flying insects the extraction of behaviorally relevant information during flight. Another possibility in order to suppress self-stimulation constitutes a corollary discharge inhibiting sensory information (e.g. [56]). However, here we argue that such an inhibitory mechanism would only impair the detection of wind generated by a bat attacking a tettigoniid that is generating syllables at a high rate. Instead we suggest that katydids avoid a self-stimulation of wind-sensilla during song production by a singing posture that increases the distance of front wings and cerci (Fig. 8). Furthermore, high-speed video observations of singing katydids showed that during stridulation the ventral margin of front wings almost stood motionless while insects were rapidly rubbing the dorsal part of their wings against each other (see Movie S1). Additionally, folded hind wings may prevent air from streaming towards cerci during stridulation. Nevertheless, further investigations are necessary in order to study self-generated wind velocities of singing katydids in detail.

### Conclusions

This is the first study suggesting that the cercal organs of katydids play an important role in the context of bat avoidance and corroborates findings obtained from neuronal response of wind-sensitive interneurons in praying mantis [5]. In contrast to praying mantis the minimum time lag between detection and contact is much longer in *T. cantans* (74 ms vs. 850 ms). This suggests that wind-sensilla enable a last moment escape maneuver from bats even when insects are busy with sound production, a behavior that impairs detection of ultrasound pulses. This has important consequences for the current understanding of acoustic advertisement signals suggesting that mate attraction is risky for the signaler, especially when predation pressure exerted by gleaning bats exploiting prey-generated sound is high [10], [12], [21]–[24], [31], [76], [77]. In such habitats, signalers often reduce their song duty cycle as a primary defense [21], [22], [31] or respond to ultrasound with song cessation as a secondary defense mechanism [12], [22], [24], [46]–[50]. Wind-sensilla as a backup sensory system may reduce predation threat exerted by bats eavesdropping acoustic advertisement signals of katydids. The next step will be to test whether tettigoniids are less successful in escaping bat predation when their cerci are ablated. This would be a direct proof of the conclusions drawn from this study.

## Materials and Methods

### Insects

*Tettigonia cantans* (*T. cantans*) and *Tettigonia viridissima* (*T. viridissima*) are closely-related species that are abundant in temperate climate zones of Europe. Both species are morphologically distinct and produce calling songs with a species-specific temporal pattern. The singing activity of both tettigoniid species usually starts in the afternoon and continues until midnight. The rhythm found within verses of *T. cantans* is monosyllabic with each syllable comprising two units, one opening hemi-syllable of low amplitude and a closing hemi-syllable of high amplitude. Verses of *T. cantans* are monosyllabic and of short duration at temperatures above 20°C [78], [79]. Syllable rate of the calling songs of *T. cantans* was 33 Hz at an ambient temperature of 21°C and 40 Hz at 28°C. In contrast the song of *T. viridissima* shows a double-syllabic structure and males sing for extended periods of time, often for half nights. The song of *T. viridissima* consists of two hemi-syllables, which are grouped into a disyllabic rhythm [57]. The double-syllable rate was 17 Hz at an ambient temperature of 28°C and 13 Hz at 21°C. Since temporal song parameters are strongly temperature dependent, we conducted ultrasound playback experiments at two different ambient temperatures (21°C and 28°C).

### Playback experiment

In order to mimic echolocation signals of gleaning bats, cosine-shaped ultrasonic pulses with a carrier frequency of 30 kHz and 10 ms duration (tapered by a rise and fall time of 3 ms) were played back to singing tettigoniids at two different pulse repetition rates (20 and 40 Hz). Total stimulus duration was limited to one second and a pause of at least 240 s was introduced after every stimulus sequence in order to avoid habituation to this stimulus. The ultrasound pulse used in playback experiments was generated in Cool Edit Pro (Syntrillium Inc.) using a sample rate of 100 kHz. Males were placed in a cage of 10×10×10 cm with walls consisting of a wire mesh (2 mm pore size). This cage was placed in a temperature controlled incubator (Ehret Inc., Type BK 4266) with walls equipped with sound absorbing foam (wedge size  = 4 cm). The temperature determined in the proximity of males was controlled with a mercury glass thermometer (B. Braun and Melsungen A.G., Germany). A custom-written macro (Spike2, Cambridge electronic design) controlled the playback of ultrasound sequences by restricting stimulus presentation to times of singing activity. For *T. cantans* males the onset of the stimulus was randomly delayed by 1–1.5 s with respect to verse onset. In total, eight *T. cantans* males and seven *T. viridissima* males were tested for their response to pulsed ultrasound stimulation in two different temperature regimes (21°C and 28°C). Playback experiments were carried out in complete darkness and lasted from 14:00 h to midnight. The singing activity of male tettigoniids was recorded with a tie-pin microphone positioned close to the male. After AD conversion (Power 1401, Cambridge electronic design) the recorded sound was stored on a personal computer (Dell Inc.).

Playback of ultrasound sequences was carried out using a DA converter (Power 1401, Cambridge electronic design) connected to a power amplifier (NAD C272, operated in mono mode). A string tweeter (Technics leaf tweeter, EAS) was positioned at a distance of 11 cm from the caged males. The sound level of 30 kHz pulses measured at the middle of the insect cage was adjusted to 89 dB SPL. European gleaning bats produce a similar amplitude (86 dB peSPL) of echolocation calls as would be measured 0.4–1.8 m distant from bats [62], [80], [81]. Stimulus intensity was calibrated by use of a sound level meter operating in “peak hold” function (CEL-414 sound level meter connected to an octave band filter CEL-296; Larson Davis microphone type 2540, serial 1898). The same measurement equipment was used to measure the level of self-generated sound at a distance one cm apart from the tympanal organ of tettigoniids.

### Neurophysiology

**Figure 9:**
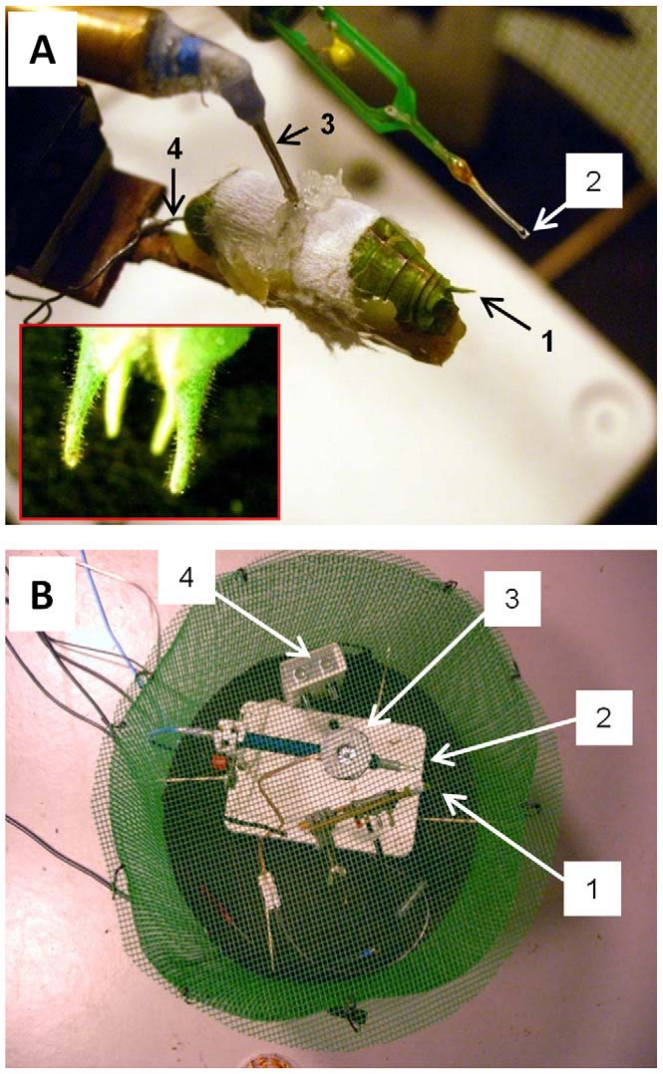
Setup for the measurement of the response of wind-sensitive interneurons to wind generated by approaching bats. (A) Close-up of an insect preparation showing the remaining cercus (1), the recording electrode (3) and indifferent electrode (4). Inset in A: The cercal apparatus of a *T. cantans* male with wind-sensilla. (B) Bats were attracted to meal worms placed on top of a wire mesh covering a loud speaker (3) broadcasting beetle rustling noise. A preparation of *T. cantans* (1) was placed close to the loudspeaker near the tip of a hot-wire anemometer (2 in A and B). Echolocation calls emitted by bats were measured by means of a technical bat detector (4).

Individuals of *T. cantans* were anesthetized with Chlorethylen and mounted to an insect holder dorsal side up using dental wax after removing head, legs and wings. A tungsten hook electrode was used to record from wind-sensitive interneurons projecting from the terminal ganglion to the thorax. These interneurons are large in diameter and receive input from wind-sensitive cercal receptors [82]. Abdominal connectives between segments 1 and 4 were surgically exposed before hooking either the left or right connective using a tungsten wire electrode. Both connectives were cut at abdominal segment 1 in order to record only ascending neuronal activity encoding the activity of wind-sensitive inter-neurons. Vaseline and paper tissue were used to prevent the nerves from drying out. The cercus contralateral to the side of the hooked connective was cut in order to prevent inhibition of nervous activity from contralateral wind-sensitive neurons [83] (Fig. 9A). Wind sensitive sensilla on an intact cercal organ of *T. cantans* are shown in the inset in figure 9A. Extracellular potentials of ascending nerve cells were amplified 1000-fold against an indifferent electrode that was inserted into the thorax. For this purpose a self-made biosignal amplifier was used, which was fabricated after Land et al. [84]. Electrode signals were digitised together with the signals of a technical bat detector (D100, Petterson Inc. Sweden) operating in frequency division mode using an AD converter (Powerlab, AD-Instruments Inc. Germany) connected to a laptop (Fujitsu Computers, Siemens). Nervous activity was manually evaluated in the software Spike2 (Cambridge Electronic Design Inc. UK).

### Flight room setup

Behavioural experiments were conducted in an indoor room (length 5.2 m, width 3.4 m, height 3 m) with walls covered with acoustic foam (Eurofoam audiotec, S230, 30 mm). This room formed the actual test arena. The insect preparation was placed one cm apart from the tip of an omni-directional hot-wire anemometer (Ahlborn, Almemo FVA 605–TA5O), which was connected to a calibrated data logger (Ahlborn, Almemo 2690) sampling the actual wind velocity every 20 ms. The preparation was positioned in the middle of the floor of the flight arena. The remaining cercus of the preparation was aligned with the long side of the flight arena. This setup allowed recording the activity of wind-sensitive neurons during the approach of *Myotis myotis*. Two adult, male individuals of this gleaning bat species were used for this study. The wing length of the bats is around 17 cm; wingspan about 40 cm. The bats were kept for behavioral experiments at the Seewiesen Max Planck Institute in an animal facility especially equipped for bat husbandry under license of the Landratsamt Starnberg (# 301c.4V-sä). They had been captured in Northern Bulgaria under license from the Ministerstvo na Okolnata Sreda I Vodita, Sofia, Bulgaria, # 1897, 16.04.2007. Bats were well habituated to the flight room and were released into the flight arena one minute before begin of the trials. In order to attract bats to the insect preparation, carabid beetle crawling sounds (see [17]) were played back from a speaker (Sennheiser, HD 555/HD 595 headphone, Part. No. 512806) placed right above the insect preparation. Additionally, decapitated mealworms were offered as a food reward right above the speaker; 6 cm above the insect preparation. Care was taken that beetle crawling sounds did not stimulate the technical bat detector. A plastic mesh with an aperture width of 5 mm covered both the insect preparation as well as the hot-wire anemometer. This mesh protected the velocity sensor from attacking bats. The bats had been trained beforehand to forage for mealworms under similar conditions. The entire setup is 36.5 cm in height and constitutes a cylinder with a diameter with 41 cm (Fig. 9B). This setup is similar to the one used by Triblehorn and Yager [5]. Flight tracks of bats were monitored with the help of three simultaneously operating video cameras (Watec, WAT-902H2) under infrared illumination. A professional video observation system was used for the storage and retrieval of video data (Abus, DigiProtect).

### Evaluation of flight paths

The influence arising from camera perspective was corrected using linear interpolation. Controls showed that the distance of the bat to the target could be determined with an accuracy of 2.1 cm. An accurate temporal alignment of video data with electrode recordings was achieved by synchronizing video frames of bat landing events with the acoustic artefact generated during landing manoeuvres of bats, which were clearly visible in the recordings of the technical bat detector.

### Ethics statement

The experiment carried out with bats investigated the wind generated by wing beats of foraging bats. This behaviour belongs to the natural behaviour repertoire of bats. Therefore, this experiment did not require an approval by an ethics committee. Bat husbandry was under license of the Landratsamt Starnberg (# 301c.4V-sä). The experiments reported in this paper comply with the current animal protection law in Austria and Germany.

### Statistics

Differences between two experimental groups was tested using a Mann Whitney Rank sum test and differences among more than two groups were tested using a Kruskal Wallis ANOVA on ranks. Differences between proportions were tested using a z-Test (with Yates correction) and correlation between parameter pairs were evaluated with a Spearman Rank Order correlation test. All statistics were calculated in Sigma Plot 11.0 (SPSS Inc.).

## Supporting Information

Movie S1 High speed videos (300 frames per second) of stridulating tettigoniids. Stridulating males appear in the following sequence: *T. cantans*, *T. viridissima* and *Mecopoda elongata* (chirping species). The latter species was filmed from two different perspectives. Males of *M. elongata* generate chirps consisting of syllables increasing in amplitude. This is reflected in the stridulatory movement of their front wings. Note that males only rub the dorsal part of both front wings against each other, whereas the tip of wings and the ventral margin of front wings almost stay motionless.
(1.87 MB MOV)
